# Role and Regulation of Mechanotransductive HIF-1α Stabilisation in Periodontal Ligament Fibroblasts

**DOI:** 10.3390/ijms21249530

**Published:** 2020-12-15

**Authors:** Christian Kirschneck, Magdalena Thuy, Alexandra Leikam, Svenja Memmert, James Deschner, Anna Damanaki, Gerrit Spanier, Peter Proff, Jonathan Jantsch, Agnes Schröder

**Affiliations:** 1 Department of Orthodontics, University Hospital Regensburg, 93053 Regensburg, Germany; magdalena.thuy@stud.uni-regensburg.de (M.T.); alexandra.leikam@stud.uni-regensburg.de (A.L.); peter.proff@ukr.de (P.P.); agnes.schroeder@ukr.de (A.S.); 2 Department of Orthodontics, University of Bonn, 53111 Bonn, Germany; Svenja.Memmert@ukbonn.de; 3 Department of Periodontology and Operative Dentistry, University of Mainz, 55131 Mainz, Germany; james.deschner@unimedizin-mainz.de (J.D.); anna.damanaki@unimedizin-mainz.de (A.D.); 4 Department of Cranio-Maxillo-Facial Surgery, University Hospital Regensburg, 93053 Regensburg, Germany; gerrit.spanier@ukr.de; 5 Institute of Clinical Microbiology and Hygiene, University Hospital Regensburg, 93053 Regensburg, Germany; jonathan.jantsch@ukr.de

**Keywords:** HIF-1α, PDLF, orthodontics, mechanotransduction

## Abstract

Orthodontic tooth movement (OTM) creates compressive and tensile strain in the periodontal ligament, causing circulation disorders. Hypoxia-inducible factor 1α (HIF-1α) has been shown to be primarily stabilised by compression, but not hypoxia in periodontal ligament fibroblasts (PDLF) during mechanical strain, which are key regulators of OTM. This study aimed to elucidate the role of heparan sulfate integrin interaction and downstream kinase phosphorylation for HIF-1α stabilisation under compressive and tensile strain and to which extent downstream synthesis of VEGF and prostaglandins is HIF-1α-dependent in a model of simulated OTM in PDLF. PDLF were subjected to compressive or tensile strain for 48 h. In various setups HIF-1α was experimentally stabilised (DMOG) or destabilised (YC-1) and mechanotransduction was inhibited by surfen and genistein. We found that HIF-1α was not stabilised by tensile, but rather by compressive strain. HIF-1α stabilisation had an inductive effect on prostaglandin and VEGF synthesis. As expected, HIF-1α destabilisation reduced VEGF expression, whereas prostaglandin synthesis was increased. Inhibition of integrin mechanotransduction via surfen or genistein prevented stabilisation of HIF-1α. A decrease in VEGF expression was observed, but not in prostaglandin synthesis. Stabilisation of HIF-1α via integrin mechanotransduction and downstream phosphorylation of kinases seems to be essential for the induction of VEGF, but not prostaglandin synthesis by PDLF during compressive (but not tensile) orthodontic strain.

## 1. Introduction

To improve dental health and aesthetics, orthodontic treatment strives to correct malpositioned teeth, as malocclusions may provoke diseases such as caries or periodontitis [1,2]. For orthodontic tooth movement (OTM), mechanical forces are applied to a tooth in the direction of movement using removable or fixed orthodontic appliances. This induces tension and pressure zones in the periodontal ligament, a connective tissue rich in collagenous fibres connecting the tooth to its surrounding alveolar bone, thereby affecting local perfusion via a compression of blood vessels [2,3]. This is accompanied by a sterile inflammatory response, which is distinguished by an increased release of cytokines and chemokines, leading to further attraction of inflammatory and immune cells into the periodontal ligament [3,4,5,6]. Other inflammatory factors such as prostaglandins (PG), predominantly PG-E2, which are produced via the inducible prostaglandin synthase 2 (PTGS-2), directly affect OTM by modulating osteoblasts and the differentiation of osteoclasts [7]. Most of the cellular and chemical adaptations occurring in pressure zones of the periodontal ligament aim to recruit osteoclast precursor cells and to differentiate them into osteoclasts that resorb the adjacent alveolar bone and remove the necrotic hyalinised tissue caused by hypoxia, thus enabling tooth movement.

Periodontal ligament fibroblasts (PDLF) comprise next to immune cells the main cell population in the periodontal ligament. PDLF are subjected to compressive or tensile mechanical strain during OTM. These cells are responsible for the remodelling of the extracellular matrix and the mediation of the inflammatory reaction underlying OTM by secretion of chemokines and interleukins. However, they also have a direct influence on bone build-up and breakdown by being able to express and secrete the receptor activator of NF-κB ligand (RANKL), while expression of the RANKL decoy receptor osteoprotegerin is reduced [3,6,8].

The adaptive response of the periodontal ligament also includes the reorganisation of the intracellular and extracellular matrix in addition to changes in local vascularisation. Mechanical strain and reduced oxygen supply are accompanied by an increased expression of hypoxia-inducible factor 1α (HIF-1α) [9], which is essentially responsible for the reoxygenation of tissue as a reaction to hypoxic conditions [10,11]. To reconstitute oxygen supply in the periodontal ligament, expression of vascular endothelial growth factor (VEGF) is enhanced after the application of compressive strain on PDLF [6,12]. A renewal of the vascular supply in compressed areas of the periodontal ligament contributes to osteoclast recruitment and differentiation; it is also an important process for healing and remodelling the periodontium. It has been shown during experimental tooth movement in rodents that cells in the periodontal ligament adjacent to hyalinised tissue and alveolar bone in pressure zones presented a substantial expression of VEGF, which is an important factor for angiogenesis [13,14]. 

Despite the observation that HIF-1α and VEGF are stabilised and upregulated during OTM in the periodontal ligament, it had been unclear whether mechanical strain itself or rather concomitant hypoxic conditions induced by blood vessel compression are the driving force for HIF-1α stabilisation and subsequent upregulation of PTGS-2, PG-E2, and VEGF expression, thus enabling OTM via increased RANKL-mediated osteoclastogenesis. A previous study showed that HIF-1α stabilisation was due rather to mechanical strain than to hypoxic conditions in PDLF in vitro [9]. Until now, however, it has been unclear how mechanotransduction of the strain applied to PDLF by orthodontic appliances is mediated leading to the biological response of HIF-1α stabilisation and to what extent the subsequent PDLF-mediated pseudoinflammatory reaction is dependent on HIF-1α stabilisation.

A possible mechanotransductive pathway and signalling could involve heparane sulfate integrin interaction and downstream phosphorylation of kinases. Cultured fibroblasts are attached to each other by focal adhesions, which are sites at which integrin receptors physically link actin-associated cytoskeletal proteins such as talin, vinculin, or paxillin with extracellular matrix components such as heparane sulfate [15], as well as with adhesion molecules on the surface of adjacent cells. Integrins are thus ideal candidates to mediate a transmission of forces across the cell membrane. Integrins function as cell adhesion molecules as well as intracellular signalling receptors, thereby mediating cell migration, proliferation, and differentiation, and also regulating intracellular signal transduction pathways [16]. The numerous signalling pathways activated by integrins are, among others, the low-molecular-weight guanosine triphosphatases Rab and Rho in mechanically stretched PDLF [17], as well as mitogen-activated protein (MAP) kinases in osteoblasts [18]. Evidence is accumulating that the previously reported HIF-1α stabilisation and associated changes in cell signalling in response to mechanical orthodontic strain are downstream of events mediated by integrins at focal adhesions. 

The aim of this study was therefore to elucidate the role of heparane sulfate integrin interaction and downstream phosphorylation of kinases for the previously observed mechanotransductive stabilisation of HIF-1α under compressive strain and to investigate whether this also occurs under tensile strain. Furthermore, we sought to investigate to what extent the downstream expression of angiogenic VEGF as well as inducible PTGS-2 and its product PG-E2 by PDLF is dependent on HIF-1α stabilisation in the context of simulated orthodontic tooth movement in vitro. This allows a better understanding of the regulation of OTM at the cellular and molecular level, which could in the future enable new therapeutic approaches by a targeted modulation of these processes.

## 2. Results

### 2.1. Strain-Dependent Stabilisation of HIF-1α

**Figure 1:**
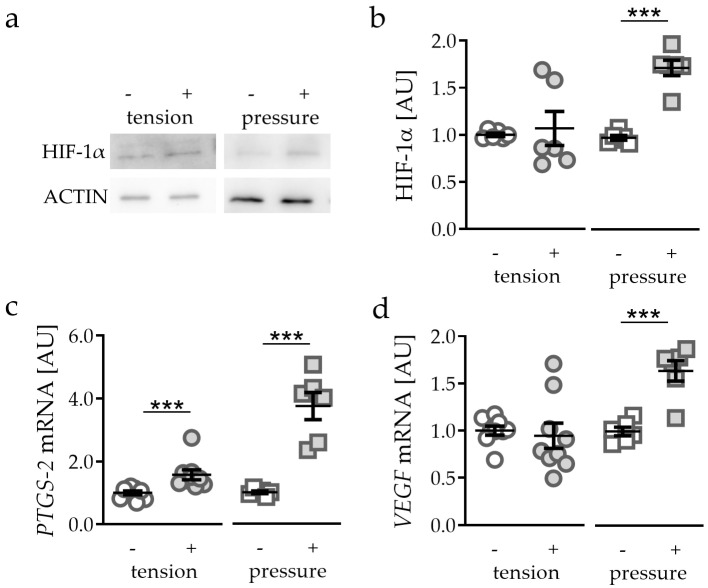
Effects of tensile strain and compressive strain on HIF-1α protein expression (**a**,**b**, n = 6) as well as *PTGS-2* (**c**) and *VEGF* (**d**) gene expression (n > 6). *Statistics:* unpaired, two-sided Mann–Whitney U or Student’s *t*-tests; AU = arbitrary units; *** *p* < 0.001.

First, we tested the effects of different mechanical strain protocols on hypoxia-inducible factor 1α (HIF-1α) protein stabilisation. Application of tensile strain failed to stabilise HIF-1α protein (*p* = 0.3939; Figure 1a,b). In contrast to tensile strain, pressure application (compressive strain) enhanced HIF-1α protein expression (*p* < 0.0001; Figure 1a,b). Analysing common HIF-1α target genes, we found a slight but significant induction of prostaglandin synthase 2 (*PTGS-2*) gene expression after tensile strain (*p* < 0.0001) and a strong induction after compressive strain (*p* < 0.0001; Figure 1c). Gene expression of vascular endothelial growth factor (*VEGF*) was not affected by tensile strain (*p* = 0.7083), while pressure application elevated *VEGF* gene expression significantly (*p* = 0.0003; Figure 1d).

### 2.2. Effects of HIF-1α Stabilisation via DMOG in PDLF during Compressive Strain

**Figure 2:**
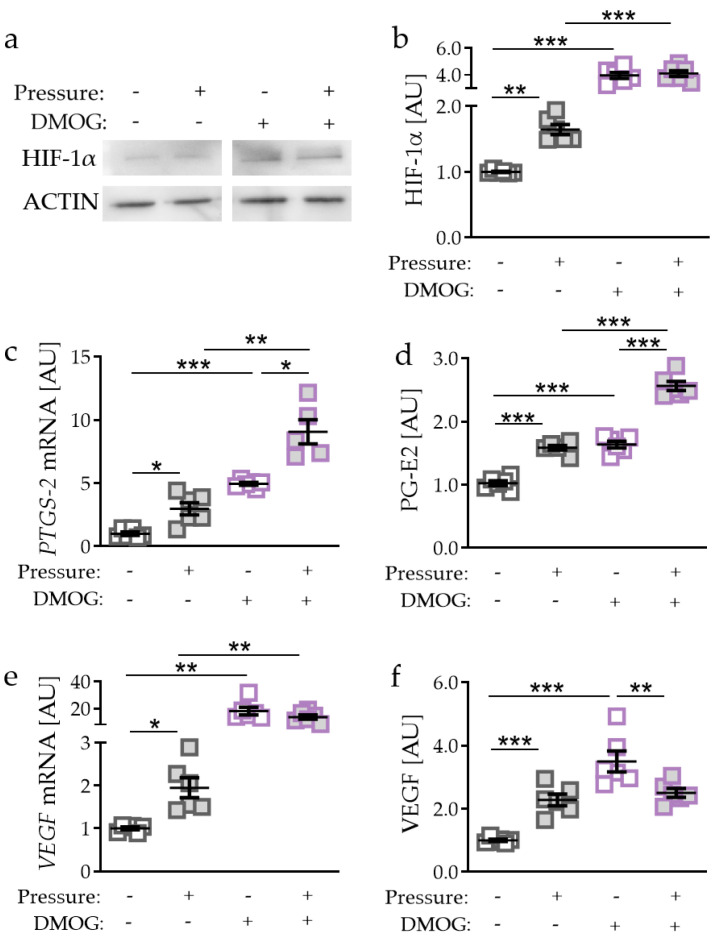
Effects of compressive strain with and without DMOG treatment (constitutively stabilising HIF-1α) on HIF-1α protein expression (**a**,**b**), *PTGS-2*/PG-E2 expression (**c**,**d**), and VEGF expression (**e**,**f**). n = 6. Statistics: ordinary one-way ANOVA followed by Holm–Sidak’s multiple comparison tests or Welch-corrected ANOVA followed by Games–Howell multiple comparison tests; AU = arbitrary units; * *p* < 0.05, ** *p* < 0.01, *** *p * < 0.001.

As we detected no effects of tensile strain on HIF-1α expression, we focused on compressive strain for the next experiments. To further investigate the role of HIF-1α during orthodontic tooth movement, we stabilised HIF-1α chemically by using DMOG (dimethyloxallyl glycine) [19]. Accordingly, we detected more HIF-1α protein with DMOG treatment without (*p* = 0.0002) and with compressive strain (*p* = 0.0002; Figure 2a,b). Of note, effects of compressive force on HIF-1α expression were truncated with DMOG treatment (*p* = 0.9709). Gene expression of *PTGS-2* was elevated by DMOG treatment in the absence (*p* < 0.0001) and presence of pressure application (*p* = 0.0052; Figure 2c). Surprisingly, we detected an even further increased *PTGS-2* gene expression with compressive strain and DMOG combined (*p* = 0.0406; Figure 2c). Accordingly, PG-E2 secretion was enhanced with DMOG-induced HIF-1α stabilisation without (*p* < 0.0001) and with compressive strain (*p* < 0.0001; Figure 2d). In line with mRNA data, PG-E2 secretion was further elevated due to pressure application under DMOG treatment (*p* < 0.0001). DMOG enhanced *VEGF* gene expression without (*p* < 0.0068) and with additional pressure application (*p* = 0.0026; Figure 2e). We did not detect any further inducible effect of pressure application on *VEGF* gene expression with simultaneous DMOG stabilisation (*p* = 0.5532). Gene expression data were confirmed by VEGF secretion. We found enhanced secretion with DMOG under control conditions without compressive strain (*p* < 0.0001; Figure 2f). VEGF secretion was downregulated with DMOG in combination with pressure application (*p* = 0.0090; Figure 2f).

### 2.3. Effects of Inhibited HIF-1α Stabilisation via YC-1 in PDLF during Compressive Strain

**Figure 3:**
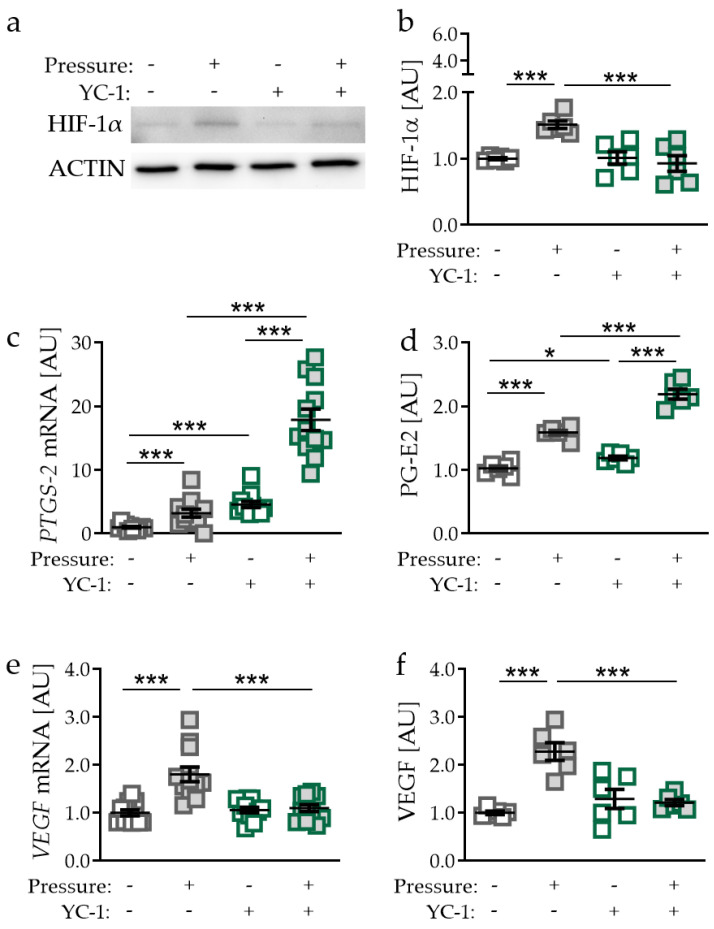
Effects of compressive strain with and without YC-1 treatment (constitutively inhibiting HIF-1α) on HIF-1α protein expression (**a**,**b**), *PTGS-2*/PG-E2 expression (**c**,**d**), and VEGF expression (**e**,**f**). Protein expression: n = 6; gene expression: n = 12. Statistics: ordinary one-way ANOVA followed by Holm–Sidak’s multiple comparison tests or Welch-corrected ANOVA followed by Games–Howell multiple comparison tests; AU = arbitrary units; * *p* < 0.05, *** *p* < 0.001.

Next, we inhibited pressure-induced HIF-1α stabilisation in PDLF using YC-1 (3-(5′-hydroxymethyl-2′-furyl)-1-benzylindazole; *p* = 0.0002; Figure 3a,b) [20]. Surprisingly, gene expression of *PTGS-2* was increased with YC-1 treatment without (*p* < 0.0001) and with compressive strain (*p* < 0.0001; Figure 3c). Even with inhibition of HIF-1α stabilisation, we detected increased *PTGS-2* gene expression when PDLF were exposed to mechanical compressive strain (*p* < 0.0001). Supporting mRNA data, PG-E2 secretion was elevated using YC-1 both in the absence (*p* = 0.0304) and in the presence of compressive strain (*p* < 0.0001; Figure 3d). Analogous to gene expression data, pressure even increased PG-E2 secretion, when HIF-1α stabilisation was inhibited by YC-1 (*p* < 0.0001; Figure 3d). Pressure application increased VEGF gene expression (*p* < 0.0001; Figure 3e). This effect was truncated by the inhibition of HIF-1a stabilisation using YC-1 (*p* < 0.0001; Figure 3e). Accordingly, we detected enhanced VEGF secretion after compressive strain (*p* < 0.0001), which was inhibited by YC-1 application (*p* < 0.0001; Figure 3f).

### 2.4. Effects of Glycosaminoglycan Antagonist Surfen on Pressure-Induced HIF-1α Stabilisation

**Figure 4:**
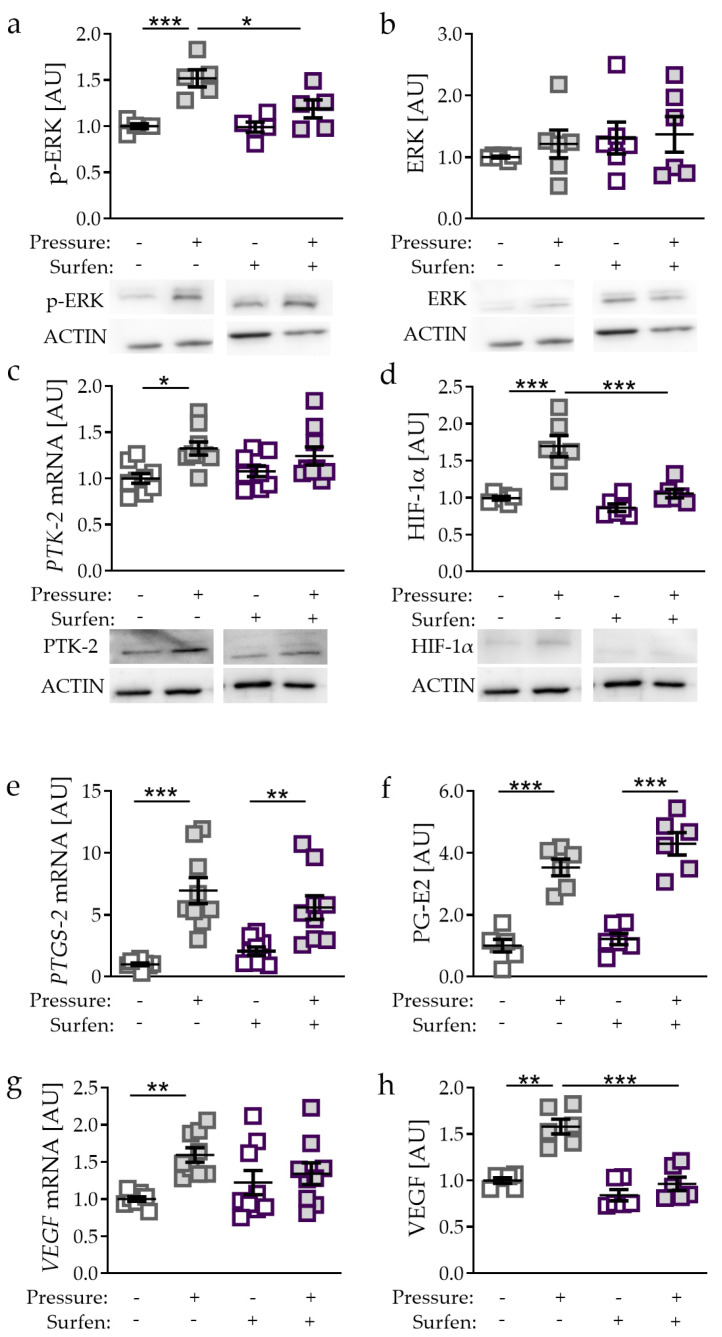
Effects of compressive strain with and without treatment with glycosaminoglycan antagonist surfen on p-ERK (**a**), ERK (**b**), PTK-2 (**c**), HIF-1α (**d**), *PTGS-2*/PG-E2 expression (**e**,**f**), and VEGF expression (**g**,**h**). Protein expression: n = 6; gene expression: n = 9. Statistics: ordinary one-way ANOVA followed by Holm–Sidak’s multiple comparison tests or Welch-corrected ANOVA followed by Games–Howell multiple comparison tests; AU = arbitrary units; * *p* < 0.05, ** *p* < 0.001, *** *p* < 0.001.

Only compressive, non-tensile strain was able to stabilise HIF-1α protein (Figure 1a,b). To further investigate the role of mechanical compressive strain on HIF-1α stabilisation in PDLF, we used the glycosaminoglycan antagonist surfen. Surfen binds glycosaminoglycans via electrostatic interaction, resulting in effective blockage of glycosaminoglycan interactions with their protein binding partners [21]. Compressive strain increased phosphorylation of extracellular signal-regulated kinase (p-ERK; *p* = 0.0007; Figure 4a), while ERK expression remained unaffected. Surfen truncated the pressure-induced effect on p-ERK protein expression significantly (*p* = 0.0241; Figure 4a). Gene and protein expression of protein-tyrosine-kinase-2 (PTK-2) was elevated upon compressive strain (*p* = 0.0114; Figure 4c) with no effect of surfen on PTK-2 expression. Next, we investigated the effect of surfen on HIF-1α and observed that surfen treatment reduced HIF-1α protein expression significantly after compressive strain (*p* < 0.0001; Figure 4d), indicating a regulatory role of glycosaminoglycan binding into HIF-1α upregulation during mechanical compressive strain. *PTGS-2* gene expression and PG-E2 secretion were both elevated by compressive strain without (both: *p* < 0.0001) and with the addition of surfen (*PTGS-2*: *p* = 0.0054; PG-E2: *p* < 0.0001; Figure 4e,f), indicating that HIF-1α is not involved in the upregulation of *PTGS-2*/PG-E2. In contrast, *VEGF* mRNA failed to be upregulated upon compressive strain when surfen was added (*p* = 0.4847; Figure 4g), whereas VEGF secretion was significantly reduced with surfen (*p* = 0.0001; Figure 4h). These data indicate that in contrast to *PTGS-2*/PG-E2, VEGF expression induced by compressive strain is controlled by HIF-1α.

### 2.5. Effects of PTK-2 Inhibitor Genistein on Pressure-Induced HIF-1α Stabilisation

**Figure 5:**
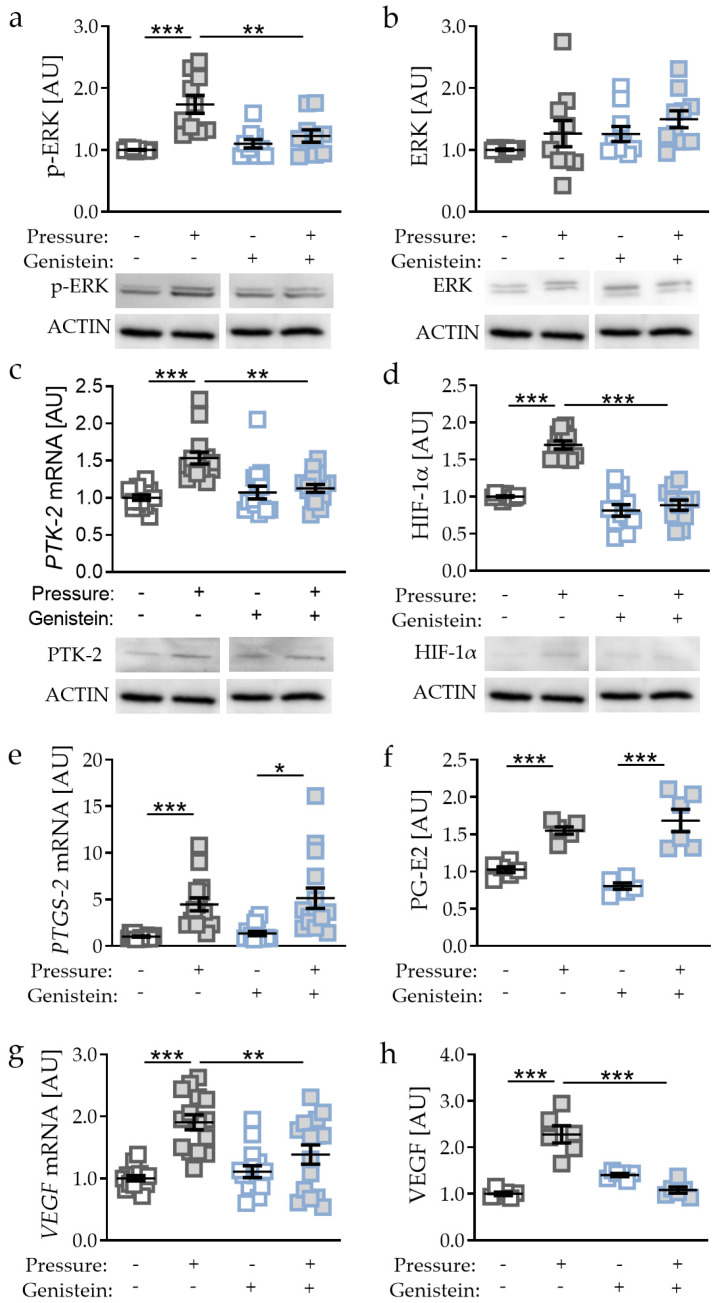
Effects of compressive strain with and without PTK-2 inhibitor genistein on p-ERK (**a**), ERK (**b**), PTK-2 (**c**), HIF-1α (**d**), *PTGS-2*/PG-E2 expression (**e**,**f**), and VEGF expression (**g**,**h**). Protein expression: n = 6; gene expression: n = 15. Statistics: ordinary one-way ANOVA followed by Holm–Sidak’s multiple comparison tests or Welch-corrected ANOVA followed by Games–Howell multiple comparison tests; AU = arbitrary units; * *p* < 0.05, ** *p* < 0.001, *** *p* < 0.001.

Genistein regulates the signal transduction component PTK-2 and thereby the phosphorylation of ERK [22,23]. Accordingly, we detected reduced p-ERK expression with genistein supplementation after pressure application (*p* = 0.0019, Figure 5a), whereas ERK protein expression was not affected by genistein (*p* = 0.7917; Figure 5b). 

As genistein is a PTK-2 inhibitor, we detected reduced PTK-2 gene and protein expression (*p* = 0.0014; Figure 5c). Inhibited PTK-2 expression and ERK phosphorylation was accompanied by reduced HIF-1α protein expression (*p* < 0.0001, Figure 5d) with genistein treatment in combination with compressive strain. Analysing HIF-1α target genes, we again detected no differences in *PTGS-2*/PG-E2 expression upon genistein-induced HIF-1α destabilisation (*PTGS-2*: *p* = 0.9548; PG-E2: *p* = 0.2669; Figure 5e,f), again supporting the previous data that *PTGS-2*/PG-E2 expression is not controlled by HIF-1α during mechanical compressive strain. In contrast, *VEGF* gene expression (*p* = 0.0061; Figure 5g) and protein secretion were reduced with genistein treatment in combination with compressive strain (*p* < 0.0001; Figure 5h), indicating that VEGF expression is controlled by HIF-1α during compressive strain in PDLF.

## 3. Discussion

In this study we demonstrated that HIF-1α is stabilised only by compressive strain, but not by tensile strain dependent on glycosaminoglycan binding to integrins at focal adhesion sites and the phosphorylation of ERK. This observation is in line with previous studies reporting a mechanotransductive stabilisation of HIF-1α during pressure application in PDLF, whereas a stabilisation of HIF-1α due to hypoxic conditions seems to play only a minor role [9]. During orthodontic tooth movement, pressure and tension zones develop in the periodontal ligament [3], resulting in reduced oxygen levels [2]. HIF-1α is the main transcriptional regulator during hypoxic conditions [10,11], regulating the transcription of hypoxia-inducible factors such as VEGF [24,25] or COX-2 [26]. Accordingly, we detected increased VEGF and COX-2 expression during pressure application, whereas only COX-2, not VEGF, was increased with tensile strain, which was previously shown in PDLF [6,27]. Our data clearly demonstrate that COX-2/PG-E2 expression during mechanical strain is HIF-1α-independent, whereas in contrast VEGF expression during compressive strain relies on HIF-1α protein stabilisation. 

VEGF is a growth factor involved in the neoformation and reorganisation of blood vessels [28]. Angiogenesis and vasodilatation of already existing blood vessels is induced during orthodontic tooth movement in the periodontal ligament at pressure zones [14]. PDLF and tendon cells increased VEGF expression after mechanical strain [6,29,30]. In a rat model, increased VEGF expression was observed after only two days of orthodontic tooth movement [31]. Improved perfusion of the periodontal ligament also enhances migration of immune cells [32], which next to PDLF are responsible for the regulation of osteoclastogenesis [33,34] and thereby for alveolar bone resorption and orthodontic tooth movement. 

Compressive strain is associated with elevated gene expression of inducible COX-2 [6,8], which catalyses the production of prostaglandins (PG), including PG-E2, from arachidonic acid [35]. Expression of COX-2 is regulated by multiple agonists such as hormones, growth factors, and proinflammatory factors. The resulting PG-E2 then mediates, amplifies, or inhibits responses to these agonists [36]. In vitro, PG-E2 was reported to regulate bone formation via osteoblast differentiation and bone resorption by controlling osteoclastogenesis. Many studies have shown that PGE2 directly stimulates osteoblastic differentiation [7] and that treatment with PG-E2 increases bone formation and bone mass in rats, mice, and humans [37,38,39]. On the other hand, PG-E2 is a potent activator of bone resorption [36,40]. Furthermore, enhanced COX-2/PG-E2 expression in osteoblastic cells contributes to an increasing osteoclast number by interleukin-1, tumor necrosis factor-α [41], and interleukin-6 [42]. PG-E2 stimulates osteoclast differentiation via upregulation of expression of receptor activator of NFκB ligand (RANKL) and inhibition of its decoy receptor, osteoprotegerin [43]. Although COX-2 was already reported to be a HIF-1α target gene [26], mechanotransductively induced expression of COX-2 seems to be HIF-1α-independent. Expression of COX-2 was reported to be alternatively activated via the transcription factor NFAT-5 (nuclear factor of activated T-cells 5) [44,45] and diverse proinflammatory stimuli [46]. 

HIF-1α protein stabilisation is not attributed only to hypoxic conditions. Ullrich et al. demonstrated that HIF-1α stabilisation in PDLF during mechanical strain was mainly due to mechanotransductive events, not hypoxia [9]. HIF-1α stabilisation was further affected by shear stress [47], osmotic stress [48], or gravity [49]. To investigate the mechanotransductive stabilisation of HIF-1α in PDLF, we inhibited glycosaminoglycan binding to integrins at focal adhesions sites, using surfen and PTK-2-induced phosphorylation of ERK using genistein. Proteoglycans such as heparane sulfate, consisting of a core protein with attached glycosaminoglycan chains, are proposed to participate in mechanotransduction in endothelial cells and fibroblasts [50,51]. Furthermore, heparane sulfate proteoglycans can act as mechanosensors in multiple cells [52] via integrins at focal adhesions sites [53]. Focal adhesion complexes are specialised sites of attachment between cells and the extracellular matrix, composed of integrins and many focal adhesion-associated cytoplasmic proteins. Because of their cellular location, focal adhesions play a role in signal transduction and have been suggested to function as mechanosensors in mature endothelial cells [54]. Surfen inhibits the interaction of proteoglycans with the focal adhesion complexes [55] and thereby truncated HIF-1α stabilisation due to mechanical strain in PDLF. These data strongly indicate an association between the interaction of proteoglycans with focal adhesion sites and mechanotransductive stabilisation of HIF-1α. Orthodontic treatment induces compressive and tensile strain in the periodontal ligament, which causes alterations within the extracellular matrix [3] and within the cytoskeleton of cells modulating the proliferation, differentiation, motility, and morphology of cells in the periodontal ligament and alveolar bone [56]. Cells are attached to the extracellular matrix by focal adhesion domains, which are multifunctional dynamic protein complexes, including integrins and cytoplasmic proteins such as the protein tyrosine kinase (PTK-2). Inhibition of PTK-2 using genistein caused reduced phosphorylation of ERK [22] and was accompanied by less HIF-1α stabilisation after compressive strain in PDLF. Our study strongly indicates that PTK-2 and the phosphorylation of ERK are involved in controlling of HIF-1α stabilisation by mechanical compressive strain during orthodontic tooth movement. 

## 4. Materials and Methods 

### 4.1. General Cell Culture Conditions

All experiments were approved by the ethics commission of the University of Regensburg (approval number 12-170-0150) and signed informed consent was obtained from all patients. Periodontal ligament fibroblasts (PDLF) were isolated and characterised as already described previously [6,57]. Briefly, periodontal connective tissue was scraped off the middle third of human teeth, digested with collagenase type II (17101-015, Thermo Fisher Scientific, Waltham, MA, USA) in phosphate-buffered saline (PBS), and cultured in DMEM high glucose (D5796, Sigma-Aldrich, St. Louis, MO, USA), supplemented with 10% FBS (P30-3306, PAN-Biotech, Aidenbach, Germany), 1% antibiotics/antimycotics (A5955, Sigma-Aldrich), 1% L-glutamine (SH30034.01, GE Healthcare, Chicago, IL, USA), and 100 µM ascorbic acid (A8960, Sigma-Aldrich, St. Louis, MO, USA) until cells proliferated out of the tissue pieces. After characterisation based on morphology and expression of fibroblast-specific genes [57], we pooled six cell lines (3 male, 3 female; aged 17–27 years) and used passage 3–6 for the experiments.

### 4.2. Reagents

**Figure 6:**
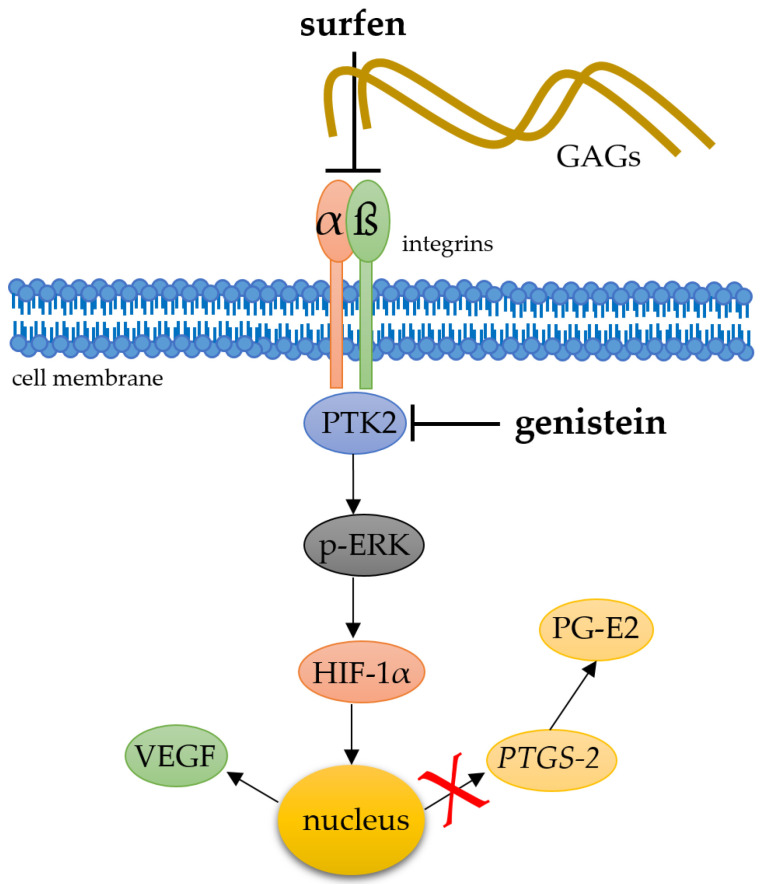
Simplified schematic presentation of a possible role of heparane sulfate integrin interaction and downstream phosphorylation of kinases for the mechanotransductive stabilisation of HIF-1α. To address this question, glycosaminoglycan antagonist surfen and PTK-2 inhibitor genistein were applied. αβ: integrins, PTK2: protein tyrosine kinase 2, p-ERK: phosphorylated extracellular signal-regulated kinases.

HIF-1α stabilisation was performed by using 500 µM DMOG (Dimethyloxallyl glycine, 71210, Cayman chemical), whereas 50 µM YC-1 (3-(5′-hydroxymethyl-2′-furyl)-1-benzylindazole) was used for the inhibition of HIF-1α stabilisation (81560, Cayman chemical). To investigate mechanotransductive integrin signalling (Figure 6), PTK-2 inhibitor genistein (G6649) and glycosaminoglycan antagonist surfen (S6951) were obtained from Sigma-Aldrich (St. Louis, MO, USA) and used in a concentration of 5 µM for the experiments.

### 4.3. Experimental Setup

Approximately 24 h before the application of mechanical compressive or tensile strain, 70,000 PDLF were either seeded on conventional polystyrene plates (353046, BD Biosciences, San Jose, CA, USA) for compressive strain or on collagen-I-coated bioflex plates (BF-3001C; Dunn Labortechnik, Asbach, Germany) for tensile strain. Reagents were applied half an hour prior to the onset of strain. PDLF were either exposed to compressive strain using sterile glass plates with 2 g/cm^2^ as described before [57,58,59] or to 16% tensile strain using spherical isotropic stamps [27] for 48 h, followed by RNA and protein extraction. Cell culture supernatant was stored at −80 °C until further use.

### 4.4. RNA Extraction

RNA extraction was performed with Trizol, which proved to ensure good RNA integrity (RIN, 28S/18S ratio) as well as the absence of genomic DNA and contamination [57]. Briefly, we carefully scraped PDLF off the plates in 1 mL PBS using a cell scraper. Cells were centrifuged at 2000 rpm for 10 min at 4 °C (HERAEUS Fresco 17 Centrifuge, Thermo Fisher Scientific, Waltham, MA, USA). The pellet was reconstituted in 500 µL peqGOLD TriFastTM (30-2010, VWR International, Radnor, PA, USA). After addition of 100 µL chloroform (1.02445.1000, VWR International, Radnor, PA, USA), samples were mixed thoroughly for a minimum of 30 s. Samples were incubated on ice for 15 min and then centrifuged for 15 min at 13,000 rpm and 4 °C. The colourless supernatant was transferred to cold 500 µL isopropanol and mixed. Samples were stored at −80 °C overnight. They were centrifuged for 30 min at 13,000 rpm at 4 °C and the pellet was washed twice with 80% ethanol in RNAse-free water (T143, Carl Roth). After drying the pellet for 30 min, it was reconstituted in 20 µL RNAse-free water (T143, Carl Roth). RNA concentration was determined using a NanoDrop photometer at 260nm (Implen, Munich, Germany).

### 4.5. cDNA Synthesis

To reduce experimental variations, we performed cDNA synthesis for all samples at once. Reverse transcription reaction was performed as described before [57] using a master mix consisting of 1x M-MLV buffer (M1705, Promega, Walldorf, Germany), 40 nmol dNTP mix (L785.2, Carl Roth, Karlsruhe, Germany), 0.1 nmol oligo-dT18 primer (SO131, Thermo Fisher Scientific, Waltham, MA, USA), 0.1 nmol random hexamer primer (SO142, Thermo Fisher Scientific, Waltham, MA, USA), 40 U RNase inhibitor (EO0381, Thermo Fisher Scientific, Waltham, MA, USA), and 200 U M-MLV reverse transcriptase (M1705, Promega, Walldorf, Germany). Samples were incubated for 1 h at 37 °C followed by inactivation of the transcriptase at 95 °C for 2 min.

### 4.6. Quantitative Real-Time Polymerase Chain Reaction (RT-qPCR)

RT-qPCR was performed as described before [57] in 96-well PCR plates (712282, Biozym Scientific, Hessisch Oldendorf, Germany) sealed with BZO Seals (712350, Biozym Scientific) in a Mastercycler^®^ ep realplex-S thermocycler (Eppendorf, Hamburg, Germany). To reduce technical errors during manual pipetting, all components except the cDNA solution were prepared as a master mix. Each reaction mix consisted of 7.5 µL SYBR^®^Green JumpStart^TM^ Taq ReadyMix^TM^ (S4438, Sigma–Aldrich, St. Louis, MO, USA), 0.375 µL forward primer, 0.375 µL reverse primer, 1.5 µL cDNA, and 5.25 µL Rnase-free H_2_O (T143, Carl Roth, Karlsruhe, Germany). After initial heat activation at 95 °C for 5 min, amplification was performed in 45 cycles (per cycle: 95 °C for 10 s, 60 °C for 8 s, 72 °C for 8 s) in duplet for each gene and biological sample. For the normalisation of target genes, we used a set of two reference genes (for compressive strain: *RPL22/PPIB*, for tensile strain: *TBP/PPIB*), which have been shown to be stably expressed in PDLF under the investigated conditions [27,57]. Cq values were defined as the second derivative maximum of the fluorescence signal curve at 521 nm and calculated with the realplex software (version 2.2, Eppendorf AG, CalqPlex algorithm). Relative gene expression was calculated as 2^−ΔCq^ [60,61] with ∆Cq = Cq (target gene) − Cq (geomean reference genes). All used primers were gene-specific and intron-flanking (Table 1) and constructed using NCBI PrimerBLAST. Primer construction and RT-qPCR quality control were performed according to the MIQE quality guidelines as described before [57,62]. Unmodified primers were synthesised and purified by Eurofins MWG Operon LLC (Huntsville, USA). For each primer pair and qPCR run a no-template-control without cDNA was included.

### 4.7. Western Blot Analysis

Protein extraction was performed using 100 µL CellLytic M (C2978, Sigma-Aldrich, St. Louis, MO, USA) per well, supplemented with proteinase and phosphatase inhibitors (4906845001, Sigma-Aldrich, St. Louis, MO, USA). Protein content was determined using RotiQuant (K015.3; Carl Roth, Karlsruhe, Germany) according to the manufacturer’s instructions. The same protein amounts were separated on a 10% Polyacrylamide gel. Proteins were blotted onto a polyvinylidene difluoride (PVDF) membrane using a tank blot system. After blocking the membrane with 5% milk (T145.3, Carl Roth) in PBS-T, the membrane was incubated with the primary antibodies anti-HIF-1α (10006421, Cayman chemicals, Ann Arbor, MI, USA, diluted 1:1000), anti-p-ERK (4370, Cell Signaling, Danvors, MA, USA, diluted 1:1000), anti-ERK (9102, Cell Signaling, Danvors, MA, USA, diluted 1:1000), anti-PTK-2 (ABIN4901515, antibodies-online, Aachen, Germany, diluted 1:1000) and anti-ACTIN (E1C602, Enogene, New York, NY, USA, diluted 1:5000) overnight at 4 °C. After three times of washing the membrane, it was incubated with horseradish-peroxidase-conjugated anti-rabbit IgG secondary antibody (1:5000, 611-1302, Rockland Immunochemicals, Gilbertsville, PA, USA) for 1 h at room temperature. After three times of washing, antibody binding was visualised with Luminata Crescendo Western HRP Substrate (WBLUR0100, Sigma-Aldrich, St. Louis, MO, USA) and recording was performed using the VWR Genoplex documentation system (VWR International, Radnor, PA, USA). Densitometric quantification of specific bands was performed with ImageJ (ver. 1.47, Wayne Rasband, National Institutes of Health, Bethesda, Maryland, USA).

### 4.8. Enzyme-Linked Immunosorbent Assays (ELISA)

Secretion of prostaglandin E2 (PG-E2) and of vascular endothelial growth factor (VEGF) protein into the PDLF cell supernatant was quantified with commercially available ELISA kits according to the manufacturers’ instructions (PG-E2: 514010, Cayman chemical, Ann Arbor, MI, USA; VEGF: RAB0507, Sigma-Aldrich, St. Louis, MO, USA). Protein expression per well was related to the respective number of PDLF, counted with a Beckman Coulter Counter (Z2 cell counter, Krefeld, Germany).

### 4.9. Statistical Analysis

Prior to the statistical analysis, all absolute data values were divided by the respective arithmetic mean of the control group without mechanical strain to obtain normalised data values relative to these controls, set to 1. Statistical analysis was performed with GraphPad Prism version 8.0 (GraphPad Software). Normal distribution was tested with a Shapiro–Wilk test. Comparing two groups, either unpaired two-tailed Mann–Whitney-U tests or Student’s *t*-tests were performed. Analysing more than two groups, Welch-corrected ANOVAs followed by Games–Howell multiple comparison tests in the case of a violation of requirements for parametric testing were performed, otherwise ordinary ANOVAs followed by Holm–Sidak’s multiple comparison tests were performed. Differences were considered significant at *p* < 0.05. The symbols in the graphs represent single data points, horizontal lines the arithmetic mean, and vertical lines the standard error of the mean.

## 5. Conclusions

Mechanotransductive stabilisation of HIF-1α in PDLF occurs with compressive strain, but not tensile strain and is dependent on glycosaminoglycan binding to integrins and regulated by PTK-2-dependent phosphorylation of ERK. An increase in VEGF but not in COX-2/PG-E2 expression during compressive strain was regulated by HIF-1α in PDLF.

## Figures and Tables

**Table 1:** Reference (*RPL22/PPIB/TBP*) and target gene primers used for RT-qPCR.

Gene Symbol	Gene Name	Accession Number	5′-Forward Primer-3′	5′-Reverse Primer-3′
*PPIB*	peptidylprolyl isomerase A	NM_000942.4	TTCCATCGTGTAATCAAGGACTTC	GCTCACCGTAGATGCTCTTTC
*RPL22*	ribosomal protein L22	NM_000983.3	TGATTGCACCCACCCTGTAG	GGTTCCCAGCTTTTCCGTTC
*TBP*	TATA-box-binding protein	NM_003194.4	CGGCTGTTTAACTTCGCTTCC	TGGGTTATCTTCACACGCCAAG
*PTGS-2*	prostaglandin endoperoxidase synthase-2	NM_000963.3	GAGCAGGCAGATGAAATACCAGTC	TGTCACCATAGAGTGCTTCCAAC
*PTK-2*	protein tyrosine kinase-2	NM_001352694.1	AGCTACAACGAGGGTGTCAAG	TGGGGCTGGCTGGATTTTAC
*VEGF*	vascular endothelial growth factor A	NM_001171623.1	TGCAGACCAAAGAAAGATAGAGC	ACGCTCCAGGACTTATACCG
